# TMT-based quantitative proteomic analysis reveals defense mechanism of wheat against the crown rot pathogen *Fusarium pseudograminearum*

**DOI:** 10.1186/s12870-021-02853-6

**Published:** 2021-02-08

**Authors:** Fangfang Qiao, Xiwen Yang, Fengdan Xu, Yuan Huang, Jiemei Zhang, Miao Song, Sumei Zhou, Meng Zhang, Dexian He

**Affiliations:** 1 College of Agronomy, Henan Agricultural University/ National Engineering Research Center for Wheat/ Co-construction State Key Laboratory of Wheat and Maize Crop Science/ Collaborative Innovation Center of Henan Grain Crops, 15 Longzihu College District, Zhengzhou, 450046 China; 2 College of Plant Protection, Henan Agricultural University, Zhengzhou, 450002 Henan China

**Keywords:** Wheat (*Triticum aestivum* L.), Crown rot, *Fusarium pseudograminearum*, TMT, Differentially expressed proteins (DEPs), Defense mechanism

## Abstract

**Background:**

Fusarium crown rot is major disease in wheat. However, the wheat defense mechanisms against this disease remain poorly understood.

**Results:**

Using tandem mass tag (TMT) quantitative proteomics, we evaluated a disease-susceptible (UC1110) and a disease-tolerant (PI610750) wheat cultivar inoculated with *Fusarium pseudograminearum* WZ-8A. The morphological and physiological results showed that the average root diameter and malondialdehyde content in the roots of PI610750 decreased 3 days post-inoculation (dpi), while the average number of root tips increased. Root vigor was significantly increased in both cultivars, indicating that the morphological, physiological, and biochemical responses of the roots to disease differed between the two cultivars. TMT analysis showed that 366 differentially expressed proteins (DEPs) were identified by Gene Ontology and Kyoto Encyclopedia of Genes and Genomes enrichment in the two comparison groups, UC1110_3dpi/UC1110_0dpi (163) and PI610750_3dpi/PI610750_0dpi (203). It may be concluded that phenylpropanoid biosynthesis (8), secondary metabolite biosynthesis (12), linolenic acid metabolites (5), glutathione metabolism (8), plant hormone signal transduction (3), MAPK signaling pathway-plant (4), and photosynthesis (12) contributed to the defense mechanisms in wheat. Protein-protein interaction network analysis showed that the DEPs interacted in both sugar metabolism and photosynthesis pathways. Sixteen genes were validated by real-time quantitative polymerase chain reaction and were found to be consistent with the proteomics data.

**Conclusion:**

The results provided insight into the molecular mechanisms of the interaction between wheat and *F. pseudograminearum*.

**Supplementary Information:**

The online version contains supplementary material available at 10.1186/s12870-021-02853-6.

## Background

Wheat (*Triticum aestivum* L.) is a major global food crop. Fusarium crown rot (FCR), caused by *Fusarium pseudograminearum*, is a major threat to wheat production. As one of the most devastating plant pathogens among soil-borne diseases, *F. pseudograminearum* can absorb nutrients from major winter cereals upon colonization [1]. The colonization of *F. pseudograminearum* initiates through epidermal penetration, most often through stomatal apertures, and progresses into the parenchymatous hypoderm. Hyphae spread from the culm base vertically through the tissues, initially through the hypoderm and pith cavity in culm tissues [2]. This pathogen mainly affects wheat, durum wheat (*Triticum turgidum* L. spp. durum (Dest.)), and barley (*Hordeum vulgare* L.) [3]. Although oats (*Avena sativa* L.) can be infected, they show few or no symptoms of disease [4]. In the Pacific Northwest and Australia, yield losses can reach up to 10–35% under natural inoculum levels [5–7]. According to reports, *F. pseudograminearum* also causes wheat crown rot in China, and in Henan, which is the largest wheat production province, the environmental conditions are especially suitable for *F. pseudograminearum.* This pathogen may present a serious threat to wheat production in the future [8]. Fully disease-resistant or immune cultivars have not been found in common wheat. Therefore, improving the genetic resistance of wheat to crown rot is an important objective.

Previous studies constructed a genetic map of *F. pseudograminearum* and completed a genomic sequence [9, 10]. Zhou et al. investigated the distribution and diversity of the pathogens associated with Fusarium crown rot in the Huanghuai wheat-growing region of China and found that *F. pseudograminearum* was the dominant species [11]. Several studies have confirmed that ER Lumenal Hsp70 protein FpLhs1, transcription factor FpAda1, and FpNPS9 are important for *F. pseudograminearum* infection [12–14]. FCR resistance responses in wheat are complex and controlled by multiple quantitative trait loci (QTLs) [15]. Thus, some studies have focused on the identification of gene loci in wheat and barley that are resistant to crown rot [16, 17]. For instance, Yang et al. used a bi-parental population derived from the wheat cultivars UC1110 and PI610750 and detected three QTLs on chromosome 6A [18]. In addition, it has been reported that *F. pseudograminearum* produces a new class of active cytokinin that could activate plant cytokinin signaling during infection [19]. These molecules may extensively reprogram the host environment through crosstalk with defense hormone signaling pathways [20, 21].

With regard to plant defense responses triggered by *F. pseudograminearum*, host–pathogen interactions have been studied by transcriptome analyses in wheat using an Affymetrix gene chip [22]. It has been suggested that the differentially expressed genes are involved in antibacterial defense, oxidative stress, and signal transduction, as well as in primary and secondary metabolism [22]. Some defense-related genes were also found to be induced more rapidly in the FCR-resistant cultivar Sunco than in the susceptible cultivar Kennedy [23]. In addition, many of the *F. pseudograminearum*-responsive genes are altered by toxin deoxynivalenol and plant defense–related hormones, which prevent *F. pseudograminearum* infection in wheat plants [22]. A combination of transcriptomics and metabolomics also has been used to study defense responses, and genes related to pathogen recognition and signal transduction, transcription factors, cell transport, and detoxification have been discovered [24].

Currently, little is known about the dynamics of the proteome and metabolome in infected host plants, and the resistance to *F. pseudograminearum* in wheat has not yet been explored from a proteomics perspective. In this study, we selected wheat cultivars with tolerance and susceptibility to *F. pseudograminearum* as the research materials. We analyzed protein expression abundance in the wheat following *F. pseudograminearum* infection using tandem mass tag (TMT) quantitative proteomics technology. Our objectives were to clarify how these proteins participate in resistance and to gather information on the inducible defense mechanisms in response to *F. pseudograminearum* infection. We also expect that this study will provide a new perspective for germplasm innovation regarding resistance to *F. pseudograminearum* in wheat, as well as contribute to the genetic improvement and breeding of new cultivars.

## Results

### Impact of *F. pseudograminearum* stress on wheat growth and development

**Fig. 1:**
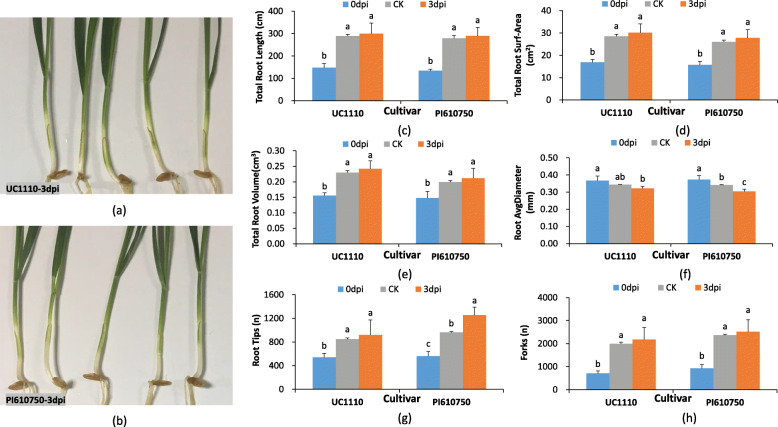
Phenotypical and morphological parameters in the response to *Fusarium pseudograminearum* infection in wheat. Data are shown as mean ± SD (*n* = 10) of three independent experiments. Different small letters (**a** or **b**) indicate a significant difference between the groups (*P* < 0.05). CK: untreated 3 dpi sample

The results showed that *F. pseudograminearum* stress affected wheat seedling growth and development, especially in the root system (Fig. 1a–h). At 3 days post-inoculation (dpi), light brown symptoms of disease initially appeared on the stem bases of the susceptible cultivar UC1110, which indicated that the incubation period was over (Fig. 1a, b). According to our observations, the average root diameter of the PI610750 seedlings was significantly decreased by 11.0% at 3 dpi compared with that of the untreated seedlings at 3 dpi (CK) (Fig. 1f), while the average number of root tips in the PI610750 seedlings was significantly increased by 30.0% (Fig. 1g). However, the total root length, total root surface area, total root volume, and forks of the UC1110 and PI610750 seedlings did not differ significantly (Fig. 1c–e, h).

**Fig. 2:**
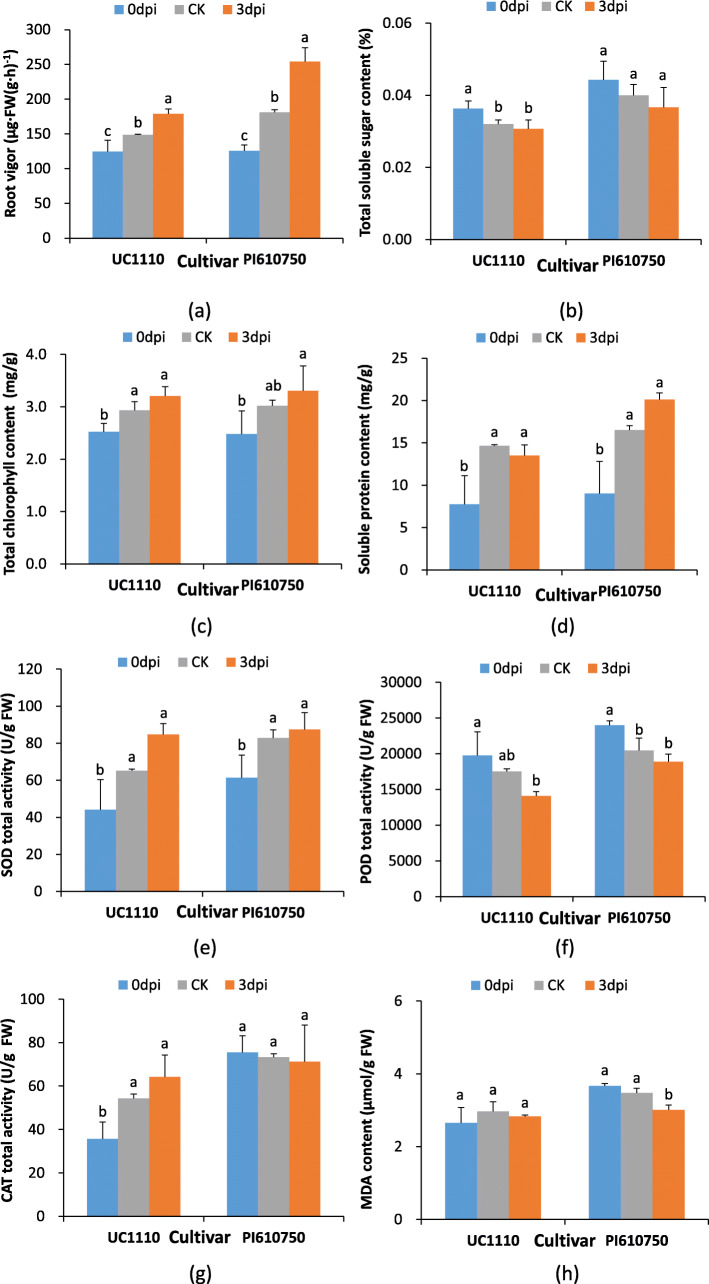
Physiological and biochemical parameters in the response to *F. pseudograminearum* infection in wheat. Data are shown as mean ± SD (*n* = 4) of three independent experiments. Different lowercase letters (**a** or **b**) indicate significant differences between the groups (*P* < 0.05). SOD: superoxide dismutase; POD: peroxidase; CAT: catalase; MDA: malondialdehyde. CK: untreated 3 dpi sample

These physiological results showed that the root vigor of both the UC1110 and PI610750 wheat seedlings was significantly increased by 20.4 and 40.5%, respectively, at 3 dpi compared with that in CK (Fig. 2a), while the malondialdehyde (MDA) content in the roots of the PI610750 seedlings was significantly decreased by 13.3% (Fig. 2h). The contents of soluble sugar and protein in the roots, the leaf chlorophyll content, and the activity of superoxide dismutase (SOD), peroxidase (POD), and catalase (CAT) in the roots of the UC1110 and PI610750 seedlings did not differ significantly (Fig. 2b–f, g). These results indicated that there were certain differences in the morphological, physiological, and biochemical responses of the disease-tolerant cultivar PI610750 and disease-susceptible cultivar UC1110 to *F. pseudograminearum* stress.

### Identification of DEPs in response to *F. pseudograminearum* infection

We comprehensively examined and identified the defense-related proteins of the stem bases of two wheat cultivars, UC1110 and PI610750, under *F. pseudograminearum* stress using TMT quantitative proteomics technology. We selected the stem bases of wheat for proteomic analysis in this experiment because they represent the first obstacle to the invasion of the crown rot pathogen *F. pseudograminearum*.

**Fig. 3:**
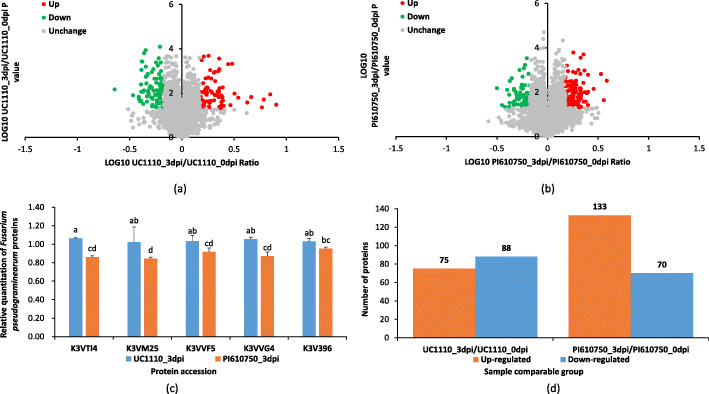
Differentially expressed proteins (DEPs) analysis between UC1110_3dpi/UC1110_0dpi and PI610750_3dpi/PI610750_0dpi. Volcano plot of all DEPs in UC1110_3dpi/UC1110_0dpi (**a**) and PI610750_3dpi/PI610750_0dpi (**b**); relative quantitation analysis of five marker proteins of *F. pseudograminearum* between UC1110_3dpi and PI610750_3dpi (**c**). Data are shown as mean ± SD (*n* = 3) of three independent experiments. Different lowercase letters (**a** or **b**) indicate significant differences between the groups (*P* < 0.05). K3VTI4: Members of the aldo keto reductase family; K3VM25: NF-X1 finger and helicase; K3VVF5: BHLH family transcription factor; K3VVG4: BHLH family transcription factor; K3V396: nucleoporin; quantitative analysis of the proteome between UC1110_3dpi/UC1110_0dpi and PI610750_3dpi/PI610750_0dpi (**d**). In blue (down-regulated): DEPs with *t*-test *P* < 0.05 and fold-change < 0.667; in orange (up-regulated): DEPs with *t*-test *P* < 0.05 and fold-change > 1.5

**Fig. 4:**
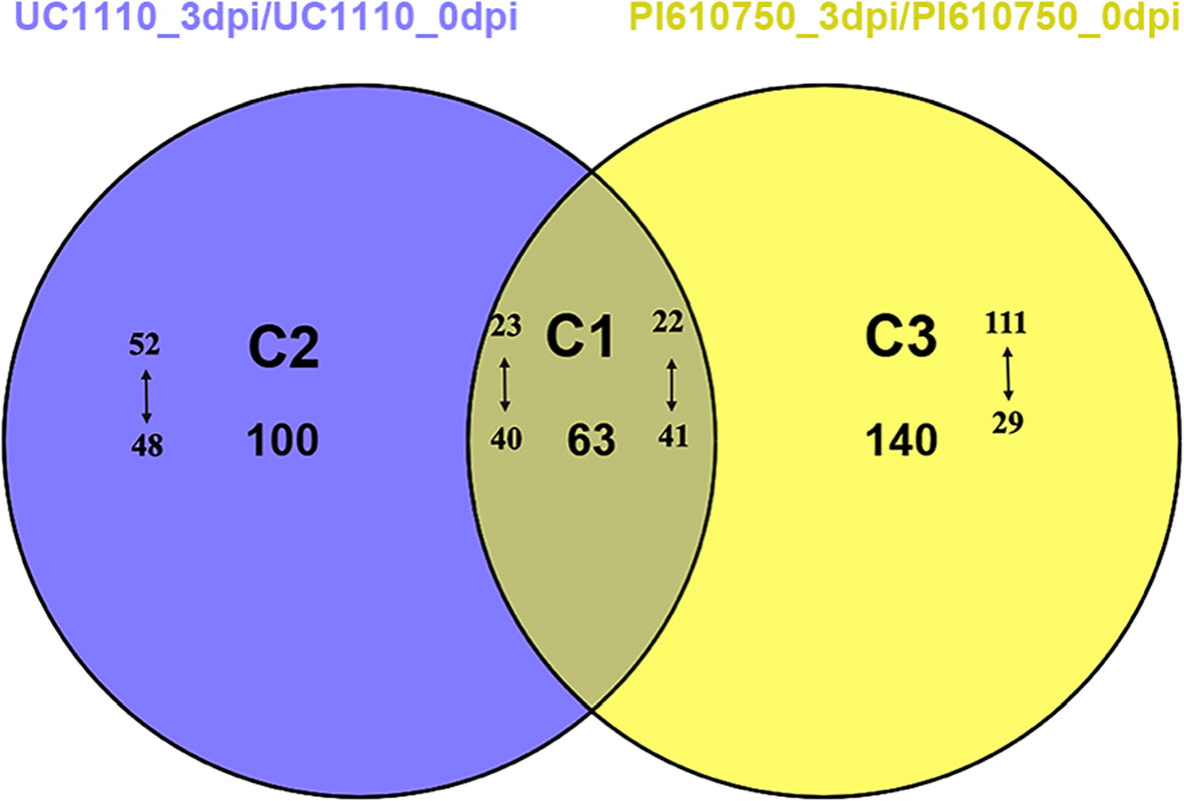
Venn diagram of the distribution of DEPs in UC1110_3dpi/UC1110_0dpi and PI610750_3dpi/PI610750_0dpi. The circles are proportional to the number of proteins identified in each treatment. The overlapping regions indicate the number of common proteins. The ↑ indicates up-regulated, while ↓ indicates down-regulated

To investigate the mechanisms of the differences in resistance of UC1110 and PI610750 at the protein level following *F. pseudograminearum* inoculation, we compared two groups, that is, UC1110_3dpi/UC1110_0dpi and PI610750_3dpi/PI610750_0dpi, using TMT quantitative proteomics. Compared with the UC1110_0dpi treatment, we identified 163 differentially expressed proteins (DEPs) in the UC1110_3dpi treatment, including 75 up-regulated and 88 down-regulated protein species, of which 100 were specifically expressed in this group (Figs. 3 and 4). In PI610750_3dpi/PI610750_0dpi, 203 protein species were differentially expressed, containing 133 up-regulated and 70 down-regulated proteins, of which 140 were specifically expressed in this group. A total of 63 proteins were common in UC1110_3dpi/UC1110_0dpi and PI610750_3dpi/PI610750_0dpi, including 23 up-regulated and 40 down-regulated proteins in UC1110_3dpi/UC1110_0dpi and 22 up-regulated and 41 down-regulated proteins in PI610750_3dpi/PI610750_0dpi (Fig. 4).

### Cluster analysis of DEPs based on GO enrichment

To determine their potential functions, we annotated 366 DEPs by Gene Ontology (GO). The GO functional enrichment analysis showed that 186, 126, and 161 protein species were enriched in biological processes, cellular components, and molecular function, respectively (Additional file 1).

#### Enrichment of DEPs related to biological processes

**Fig. 5:**
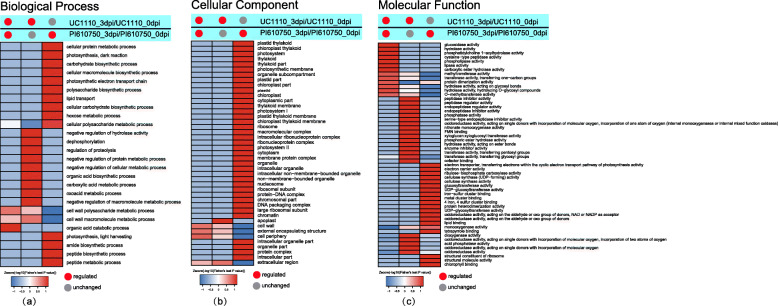
GO-functional enrichment cluster analysis of DEPs: (**a**) biological process enrichment analysis; (**b**) cellular component enrichment analysis; (**c**) molecular functional enrichment analysis

Under biological processes, the common DEPs (C1) in the two comparison groups of UC1110_3dpi/UC1110_0dpi and PI610750_3dpi/PI610750_0dpi were significantly enriched in the terms of organic acid catabolism, cell wall polysaccharide metabolism, and cell wall macromolecule metabolism (Fig. 5a). The specific DEPs (C2) of UC1110_3dpi/UC1110_0dpi were significantly enriched in the terms of negative regulation of hydrolase activity, dephosphorylation, regulation of proteolysis, negative regulation of protein metabolism, negative regulation of cellular metabolism, organic acid biosynthesis, carboxylic acid metabolism, oxoacid metabolism, and negative regulation of macromolecule metabolism (Fig. 5a). The specific DEPs (C3) of PI610750_3dpi/PI610750_0dpi were significantly enriched in the terms of cellular protein metabolism, photosynthesis (dark reaction), carbohydrate biosynthesis, cellular macromolecule biosynthesis, the photosynthetic electron transport chain, polysaccharide biosynthesis, lipid transport, cellular carbohydrate biosynthesis, hexose metabolism, cellular polysaccharide metabolism, photosynthesis (light harvesting), amide biosynthesis, peptide biosynthesis, and peptide metabolism (Fig. 5a). This analysis showed that the DEPs related to organic acid catabolism and cell wall metabolism responded to *F. pseudograminearum* stress in the seedling stem bases of both the susceptible and tolerant cultivars. The disease-susceptible cultivar UC1110 also responded to stress through the DEPs related to dephosphorylation and carboxylic acid metabolism, while the disease-tolerant cultivar PI610750 mainly responded to stress through the DEPs related to photosynthesis and sugar metabolism.

#### Enrichment of DEPs related to cellular components

In the cellular components category, the common DEPs (C1) in the two comparison groups of UC1110_3dpi/UC1110_0dpi and PI610750_3dpi/PI610750_0dpi were significantly enriched in the cell walls, external encapsulating structures, the cell periphery, and extracellular regions (Fig. 5b). The specific DEPs (C2) of UC1110_3dpi/UC1110_0dpi were significantly enriched in the apoplasts (Fig. 5b). The specific DEPs (C3) of PI610750_3dpi/PI610750_0dpi were significantly enriched in the plastid thylakoids, chloroplast thylakoids, photosystem, photosynthetic membranes, cytoplasmic parts, thylakoid membranes, ribosomes, ribonucleoprotein complex, cytoplasm, membrane protein complex, and organelles (Fig. 5b). This analysis showed that the DEPs related to the cell wall first responded to *F. pseudograminearum* stress in the seedling stem bases of both the susceptible and tolerant cultivars. The disease-susceptible cultivar UC1110 also responded to stress through the DEPs related to apoplasts, while the disease-tolerant cultivar PI610750 mainly responded through the DEPs related to chloroplasts.

#### Enrichment of DEPs related to molecular function

In terms of molecular function, the common DEPs (C1) in the two comparison groups of UC1110_3dpi/UC1110_0dpi and PI610750_3dpi/PI610750_0dpi were significantly enriched in the terms of glucosidase activity, hydrolase activity, phosphatidylcholine1-acylhydrolase activity, cysteine-type peptidase activity, phospholipase activity, lipase activity, and carboxylic ester hydrolase activity (Fig. 5c). The specific DEPs (C2) of UC1110_3dpi/UC1110_0dpi were significantly enriched in the terms of the peptidase regulator activity, endopeptidase regulator activity, phosphatase activity, oxidoreductase activity, nitronate monooxygenase activity, flavin mononucleotide binding, hydrolase activity, enzyme inhibitor activity, transferase activity, dioxygenase activity, and acid phosphatase activity (Fig. 5c). The specific DEPs (C3) of PI610750_3dpi/PI610750_0dpi were significantly enriched in the terms of the electron transporter activity, electron carrier activity, ribulose-bisphosphate carboxylase activity, cellulose synthase activity, glucosyltransferase activity, metal cluster binding, protein heterodimerization activity, oxidoreductase activity, lipid binding, tetrapyrrole binding, structural constituents of ribosomes, structural molecule activity, and chlorophyll binding (Fig. 5c). This analysis showed that the disease-tolerant cultivar PI610750 mainly responded to stress through the DEPs related to electron transporter activity, electron carrier activity, cellulose synthase activity, and oxidoreductase activity in the plant-pathogen interaction process.

### Kyoto encyclopedia of genes and genomes pathway enrichment analysis of DEPs

**Fig. 6:**
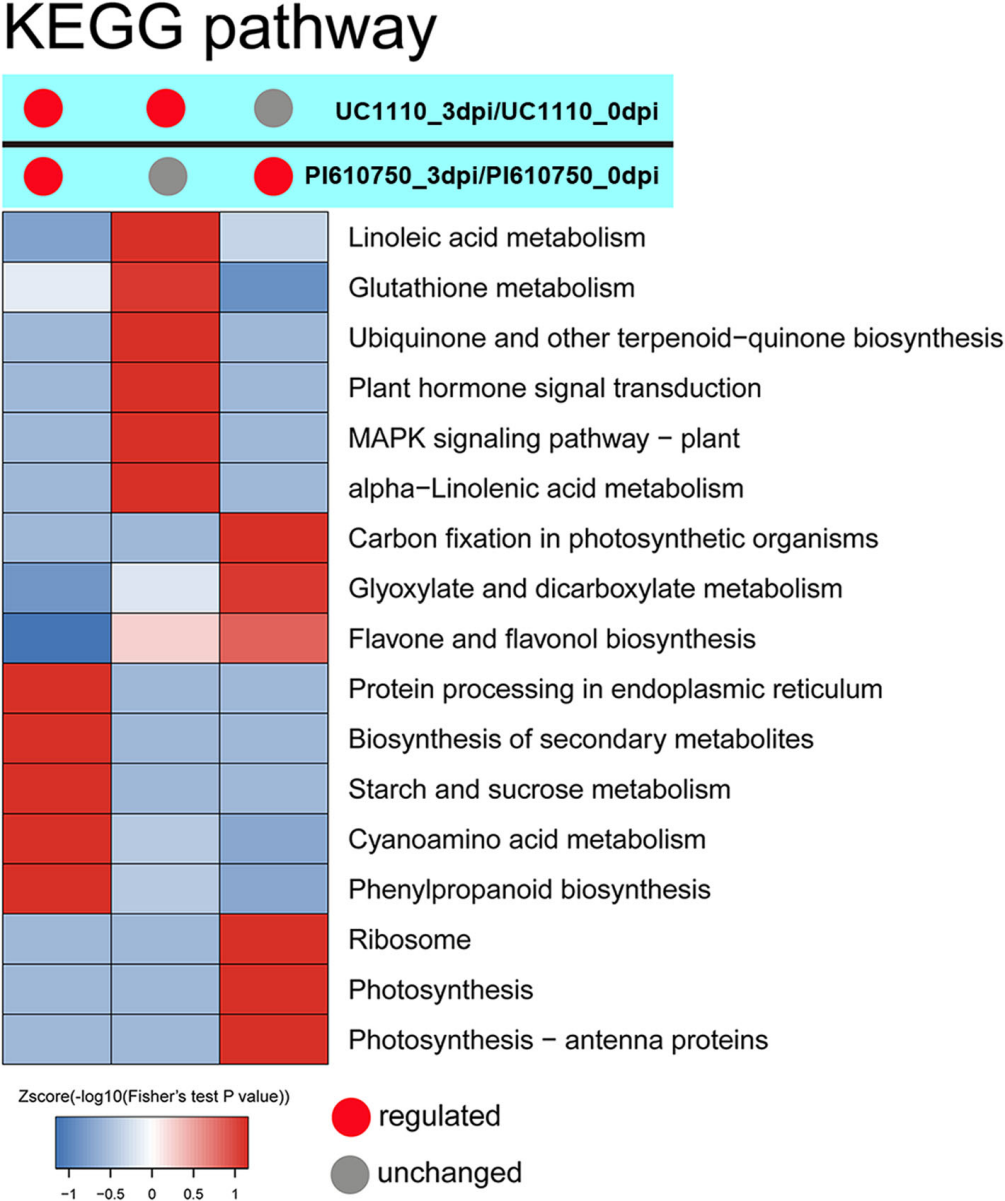
KEGG pathway enrichment cluster analysis of the DEPs of the two comparison groups

Further analysis using Kyoto Encyclopedia of Genes and Genomes (KEGG) pathway enrichment showed that all DEPs of the two comparison groups were significantly enriched in the terms of ribosomes (20%), phenylpropanoid biosynthesis (14%), photosynthesis (11%), glutathione metabolism (11%), carbon fixation in photosynthetic organisms (7%), alpha-linolenic acid metabolism (7%), glyoxylate and dicarboxylate metabolism (7%), linoleic acid metabolism (6%), cyanoamino acid metabolism (6%), photosynthesis-antenna proteins (6%), and flavone and flavonol biosynthesis (5%) (Additional file 2). The 63 common DEPs (C1) in the two comparison groups of UC1110_3dpi/UC1110_0dpi and PI610750_3dpi/PI610750_0dpi were significantly enriched in the terms of biosynthesis of secondary metabolites (osa01110), phenylpropanoid biosynthesis (osa00940), protein processing in endoplasmic reticulum (osa04141), starch and sucrose metabolism (osa00500), and cyanoamino acid metabolism (osa00460) (Fig. 6, Additional file 3).

The specific DEPs (C2) of UC1110_3dpi/UC1110_0dpi were significantly enriched in the terms of linoleic acid metabolism (osa00591), glutathione metabolism (osa00480), alpha-linolenic acid metabolism (osa00592), MAPK signaling pathway-plant (osa04016), flavone and flavonol biosynthesis (osa00944), and ubiquinone and other terpenoid-quinone biosynthesis (osa00130) (Fig. 6, Additional file 4). The specific DEPs (C3) of PI610750_3dpi/PI610750_0dpi were significantly enriched in the terms of ribosomes (osa03010), photosynthesis (osa00195), photosynthesis-antenna proteins (osa00196), flavone and flavonol biosynthesis (osa00944), carbon fixation in photosynthetic organisms (osa00710), and glyoxylate and dicarboxylate metabolism (osa00630) (Fig. 6, Additional file 5). This analysis showed that the DEPs related to secondary metabolites, protein processing, and energy metabolism pathways responded to stress in the seedling stem bases of both the susceptible and tolerant cultivars. The susceptible cultivar UC1110 responded to stress mainly through the DEPs related to linoleic acid metabolism and glutathione metabolism, and the disease-tolerant cultivar PI610750 mainly responded through the DEPs related to photosynthesis and glyoxylic acid and dicarboxylate metabolism.

### Interaction network analysis of *F. pseudograminearum*-responsive proteins in wheat

**Fig. 7:**
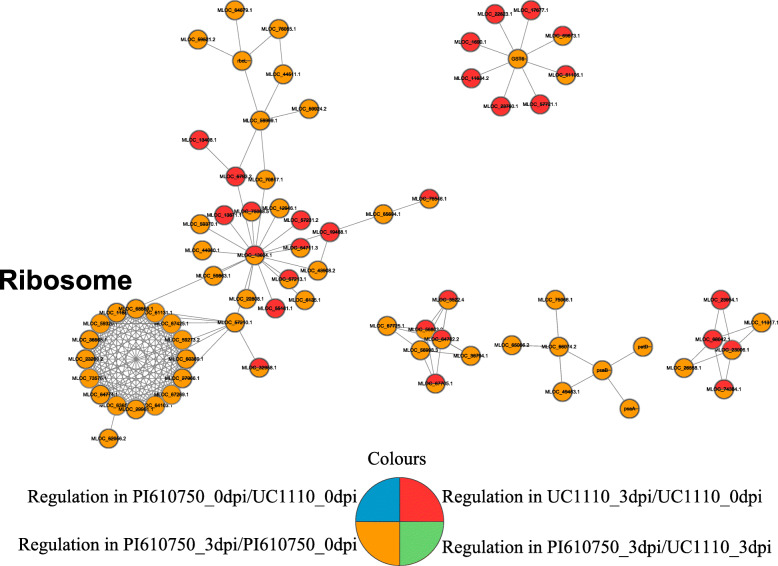
Protein-protein interaction network analysis of the DEPs of the two comparison groups

The present study used the online STRING database and Cytoscape software to construct a protein-protein interaction network for all DEPs of the two comparison groups in response to *F. pseudograminearum*. This network showed that 76 of the possible DEPs interacted. With the MCODE plug-in toolkit, three enriched interaction clusters were associated with ribosomes, photosynthesis, and sugar metabolism (Fig. 7). Sixteen interaction proteins belonged to the ribosome network. These proteins included 15 up-regulated proteins and one down-regulated protein in the comparison group PI610750_3dpi/PI610750_0dpi. Seven interaction proteins belonged to the carbohydrate metabolic process network, including four down-regulated proteins in the two comparison groups of UC1110_3dpi/UC1110_0dpi and PI610750_3dpi/PI610750_0dpi, and one down-regulated and two up-regulated proteins in the comparison group of PI610750_3dpi/PI610750_0dpi. Three interaction proteins belonged to the photosynthesis network, including three up-regulated proteins in PI610750_3dpi/PI610750_0dpi. Further information about the proteins is shown in Additional file 6.

### Correlation between mRNA and protein abundance

To further validate the reliability of the proteomics data, we selected 16 genes for quantitative real time–polymerase chain reaction (qRT-PCR) analysis. Three common genes in the two comparison groups showed similar tendencies as those for protein expression, including NMT1, GLU1B, and XIPI. In the comparison group of UC1110_3dpi/UC1110_0dpi, NMT1 and GLU1B were up-regulated and down-regulated, respectively. In the comparison group of PI610750_3dpi/PI610750_0dpi, however, both XIPI and GLU1B were down-regulated (Table 1). In the comparison group of UC1110_3dpi/UC1110_0dpi, five specific DEPs were up-regulated at both the transcription and translation levels. Similarly, in the comparison group of PI610750_3dpi/PI610750_0dpi, eight specific DEPs were up-regulated at both the transcription level and translation level. The primer sequences for the 16 genes are listed in Additional file 7.

**Table 1:** The mRNA and protein abundance changes of 16 selected genes in the study

Gene names	UC1110-3dpi vs UC1110-0dpi				PI610750-3dpi vs PI610750-0dpi			
	Gene fold changes	Regulated type	Protein fold changes	Regulated type	Gene fold changes	Regulated type	Protein fold changes	Regulated type
GLU1B	0.25	Down	0.46	Down	0.15	Down	0.48	Down
NMT1	2.70	Up	1.68	Up	0.74	Down	1.78	Up
XIPI	1.46	Up	0.58	Down	0.34	Down	0.64	Down
cla30	2.41	Up	2.25	Up	–	–	–	–
gstu2	15.48	Up	2.27	Up	–	–	–	–
Pr-1-2	4.18	Up	7.00	Up	–	–	–	–
Pr-1-1	8.43	Up	6.08	Up	–	–	–	–
PR-1.2	29.84	Up	2.35	Up	–	–	–	–
LIM	–	–	–	–	2.21	Up	1.69	Up
psaC	–	–	–	–	1.15	Up	2.13	Up
petD	–	–	–	–	1.19	Up	1.66	Up
rps11	–	–	–	–	1.94	Up	1.56	Up
CENH3	–	–	–	–	2.44	Up	1.73	Up
ltp9.4b	–	–	–	–	1.81	Up	1.51	Up
TRAES_3BF087500010CFD_c1	–	–	–	–	1.10	Up	2.51	Up
TRAES_3BF088300010CFD_c1	–	–	–	–	1.21	Up	2.51	Up

## Discussion

The roots of the two wheat cultivars with different levels of disease tolerance showed obvious differences in their morphological, physiological, and biochemical responses to the crown rot pathogen *F. pseudograminearum*. Average root diameter and MDA content in the roots of the PI610750 seedlings decreased, while the average number of root tips increased. The increase in root vigor was higher at 3 dpi in PI610750 than in UC1110. This indicated that the defense mechanism of the wheat seedlings to the crown rot pathogen *F. pseudograminearum* was complex. Although many factors related to crown rot resistance have been identified, the molecular mechanisms of crown rot resistance are still poorly understood. Therefore, understanding the defense mechanism of wheat plants against crown rot is crucial to the sustainable improvement of wheat yield and quality. The DEPs of the two cultivars in this study were associated with metabolic pathways, plant-pathogen interaction, and photosynthesis.

### Metabolic pathways in response to *F. pseudograminearum* infection

The metabolic pathways of the response of wheat to *F. pseudograminearum* were essential, accounting for 37% of DEPs in all KEGG pathways. Previous studies have shown that proline metabolism is implicated in the plant response to abiotic stress, and proline dehydrogenase (ProDH) is the first enzyme to catalyze the degradation of proline [25]. According to reports, 4-hydroxy-7-methoxy-3-oxo − 3,4-dihydro-2H-1,4-benzoxazine-2-yl glucoside β-D-glucosidase is a typical member of multiple metabolic pathways, as 4-hydroxy-7-methoxy-3-oxo-3,4-dihydro-2H-1,4-benzoxazin-2-yl glucoside β-D-glucosidase is involved in the metabolism of high-energy compounds and plant growth [26]. Some studies have shown that β-glucosidase is involved in catalyzing the hydrolysis of glycosides to release glucose into the glycolysis process [27]. In plants, glutathione S-transferases (GSTs) have been shown to play a major role in cell detoxification and stress tolerance [28–30]. Previously, it was reported that lipoxygenase, allene oxide cyclase, and allene oxide synthase (AOS) are three important enzymes in jasmonic acid (JA) biosynthesis, and the activation of AOS enhances the drought tolerance of chickpea [31–33]. Studies have also shown that AOS transcripts and JA concentration in cells are critical for responses to pathogen and/or virus infections in plants [34, 35].

Some studies have indicated that NADH-dependent glutamate synthetase (NADH-GOGAT) is located in non-green tissues and is highly expressed in the roots, participates in the ammonium assimilation pathway, and promotes the absorption of nitrogen by plants [36]. Previous research has shown that the two enzymes phenylalanine ammonia lyase and cinnamoyl-CoA reductase are mainly involved in lignin biosynthesis. The biosynthesis of lignin is a major branch in the phenylpropane biosynthesis pathway, and the biosynthesis of phenylpropane is involved in the resistance of plants to diseases [37–39]. In this study, we found five common DEPs in the two comparison groups of UC1110_3dpi/UC1110_0dpi and PI610750_3dpi/PI610750_0dpi to be significantly down-regulated, including ProDH, 4-hydroxy-7-methoxy-3-oxo-3,4-dihydro-2H-1,4-benzoxazin-2-yl glucoside beta-D-glucosidase, beta-glucosidase 26, AOS, and GSTU1. ProDH, AOS, and GSTU1 were enriched in the KEGG pathways of arginine and proline metabolism (osa00330), alpha-linolenic acid metabolism (osa00592), and glutathione metabolism (osa00480), respectively.

**Fig. 8:**
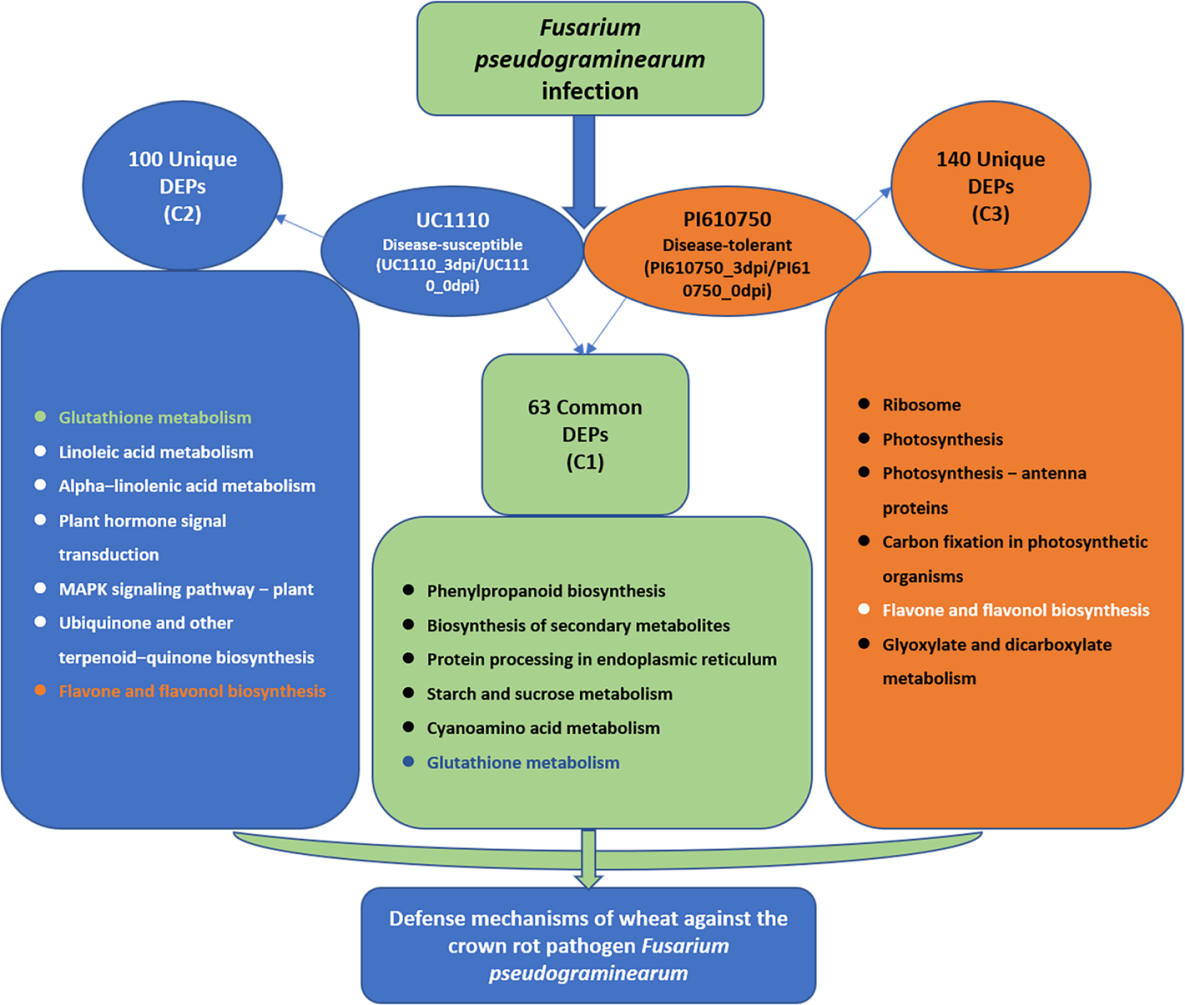
Schematic model of the defense mechanisms of wheat against the crown rot pathogen *F. pseudograminearum*

In addition, 4-hydroxy-7-methoxy-3-oxo-3,4-dihydro-2H-1,4-benzoxazin-2-yl glucoside beta-D-glucosidase, and beta-glucosidase 26 were enriched in the three pathways of starch and sucrose metabolism (osa00500), cyanoamino acid metabolism (osa00460), and phenylpropanoid biosynthesis (osa00940) in the two comparison groups UC1110_3dpi/UC1110_0dpi and PI610750_3dpi/PI610750_0dpi. GSTU6 and NADH-GOGAT were up-regulated and were enriched in the pathways of glutathione metabolism (osa00480) and nitrogen metabolism (osa00910), respectively. Cinnamoyl-CoA reductase 1, peroxidase, and phenylalanine ammonia-lyase were also up-regulated and were enriched in the pathway of phenylpropanoid biosynthesis (osa00940). This indicated the following: (1) the ProDH and 4-hydroxy-7-methoxy-3-oxo-3,4-dihydro-2H-1,4-benzoxazine-2-yl glucoside βD-glucoside enzymes play an important role in the defense mechanism of wheat against *F. pseudograminearum*; (2) the study of GSTs in these two wheat cultivars may reveal the differences in the role of GSTU1 and GSTU6 in the defense mechanism of wheat against *F. pseudograminearum*; (3) the down-regulation of AOS in the JA pathway makes UC1110 more susceptible to pathogen infection; (4) cinnamoyl-CoA reductase 1, peroxidase, and phenylalanine ammonia lyase were up-regulated in the biosynthesis of phenylpropane, which is highly related to plant defense ability; and (5) the metabolic pathways of plants in response to pathogenic stress are complex and changeable (Fig. 8).

### Plant-pathogen interactions in wheat

Plants have various defense mechanisms. These include the production of antimicrobial peptides, particularly pathogenesis-related proteins (PR proteins). PR proteins were first noted in plants as part of the hypersensitive response, but have since been assigned an array of biological roles [40]. PR proteins are a type of stress-responsive protein whose expression can be induced by pathogen invasion [41]. A number of studies have shown that PR proteins participate in plant defense mechanisms, as many of them are endowed with antimicrobial activity against plant pathogens, with different antifungal, antibacterial, and antiviral effects [42, 43]. Regarding the specific DEPs in the comparison group of UC1110_3dpi/UC1110_0dpi, multiple PR proteins related to plant-pathogen interactions were identified, including PR protein-1.2, PR protein 1–1, and PR protein 1–2. The expression levels of these proteins were up-regulated in response to *F. pseudograminearum* infection. These proteins were up-regulated in PI610750_3dpi/PI610750_0dpi, but the difference was not significant.

PR protein 1 is an antimicrobial protein in host defense that is targeted by plant pathogens during infection [44–46]. The production of PR proteins in response to pathogen invasion is related to the plant disease resistance specialized in systemic acquired resistance (SAR) [47]. PR-1-5 is a potential target of ToxA, and the site-specific interaction between PR-1-5 and ToxA may mediate ToxA-induced necrosis of susceptible wheat [48]. In this study, pathogenesis-related (PR-1.2, Pr-1-1, Pr-1-2) proteins were up-regulated in the comparison group of UC1110_3dpi/UC1110_0dpi and were involved in plant hormone signal transduction (osa04075) and the MAPK signal plant pathway (osa04016), suggesting that the PR1 family plays an important role in the crown rot defense mechanism of wheat.

### Defensive photosynthetic activities of wheat stem bases under *F. pseudograminearum* infection

In plants, chloroplast photosynthesis is an important biochemical reaction that converts light energy into chemical energy to maintain plant life [49]. Research on plant defense and photosynthesis has indicated that the rate of photosynthesis is reduced after pathogen invasion, such as in barley infected with powdery mildew, potato infected with *Phytophthora infestans*, and soybean infected with *Phytophthora sojae* [50–52]. In this study, the specific DEPs in the comparison group of PI610750_3dpi/PI610750_0dpi were significantly enriched in the three photosynthesis-related pathways of photosynthesis (osa00195), photosynthesis-antennary protein (osa00196), and carbon fixation (osa00710) (Fig. 6). The increase in the abundance of photosynthesis-related proteins may reflect the fact that photosynthesis provides a large amount of energy for plant defense. Thus, photosynthesis-related proteins in the disease-tolerant cultivar PI610750 play an important role in disease defense.

Previous studies have shown that sedoheptulose-1,7-bisphosphatase, phosphoribulokinase, glyceraldehyde-3-phosphate dehydrogenase, and ribulose 1,5-bisphosphate carboxylase/oxygenase (Rubisco) are involved in the Calvin cycle, and phosphoglycerate kinase participates in the glycolytic, gluconeogenic, and photosynthetic pathways [53, 54]. It also has been reported that increasing the activity of sedoheptulose-1,7-bisphosphatase in transgenic tobacco plants can promote photosynthesis and growth from the early stages of development [55]. Rubisco is an enzyme complex in plants that is composed of eight large subunits and eight small subunits [56]. It has been reported that the abundance of the small and large subunits of Rubisco increased significantly in Zhongmu-1 8 h after salt treatment [57]. An increase in the abundance of Rubisco large subunits and a decrease in small subunits have also been detected in nontransgenic wheat in response to drought [58, 59]. It has been reported that Rubisco large subunits and ribose-1 are down-regulated at 24 h post-inoculation and then up-regulated at 48 and 72 h post-inoculation [60].

In our study, we detected enriched sedoheptulose-1,7-bisphosphatase, phosphoribulokinase, phosphoglycerate kinase, glyceraldehyde-3-phosphate dehydrogenase, Rubisco large subunits, and Rubisco small subunits in the carbon fixation pathway of photosynthetic organisms (osa00710) of PI610750. The increase in abundance of these DEPs indicated that photosynthesis plays a major role in the defense mechanisms of the disease-tolerant cultivar PI610750. However, these DEPs were not observed in the disease-susceptible cultivar UC1110. In summary, the disease-tolerant cultivar PI610750 may defend itself against disease by increasing its photosynthetic rate, thus providing energy for itself.

## Conclusions

Through TMT-based quantitative proteomic analysis, we confirmed that the physiological and biochemical responses of the wheat disease-tolerant cultivar PI610750 and disease-susceptible cultivar UC1110 were significantly different under *F. pseudograminearum* stress. Based on the cluster analysis results of the GO enrichment and KEGG pathway enrichment, the metabolic pathways of the wheat response to *F. pseudograminearum* stress may be complex. The disease-tolerant cultivar PI610750 and the susceptible cultivar UC1110 interacted with pathogens during the incubation period. Although these cultivars shared many of the same metabolic pathways, they also possessed unique pathways. The unique pathways in the susceptible cultivar UC1110 were mainly related to linoleic acid metabolism, plant hormone signal transduction, MAPK signaling pathway–plant, and ubiquinone biosynthesis, while the unique pathways in the disease-tolerant cultivar PI610750 were mainly related to photosynthesis, carbon fixation in photosynthetic organisms, flavone and flavonol biosynthesis, and glyoxylate and dicarboxylate metabolism. The DEPs in the seedling stem bases of the disease-susceptible cultivar UC1110 were mainly related to glutathione metabolism, nitrogen metabolism, and phenylpropane biosynthesis, whereas the DEPs in the seedling stem bases of the disease-tolerant cultivar PI610750 were mainly related to photosynthesis. This indicated that there are differences in the defense mechanism of the disease-tolerant wheat cultivar PI610750 and the disease-susceptible cultivar UC1110 against *F. pseudograminearum*, which might provide a perspective for wheat genetic improvement and breeding.

## Methods

### Experimental materials and inoculation

The wheat cultivars UC1110 and PI610750, which were kindly provided by Prof. Jorge Dubcovsky from the University of California, Davis, were used in our experiments. A strong and aggressive *F. pseudograminearum* strain, WZ-8A (Accession: JN862232.1), which was kindly provided by Prof. Honglian Li from the College of Plant Protection of Henan Agricultural University, was used in this study. The cultivar UC1110 is susceptible to the predominant Chinese isolate WZ-8A of *F. pseudograminearum*, whereas the cultivar PI610750 is tolerant. UC1110 and PI610750 seeds were sterilized by immersion in 75% (w/v) alcohol for 30 s and then thoroughly washed with distilled water. The sterilized seeds were cultivated in sterilized pots (12 cm × 17 cm) with 2 kg sterilized soil (sand: soil = 2.5:1). The seedlings were maintained in a growth chamber at 25/20 °C day/night temperatures under a 16/8 h light/dark photoperiod and 65/75% day/night relative humidity.

**Fig. 9:**
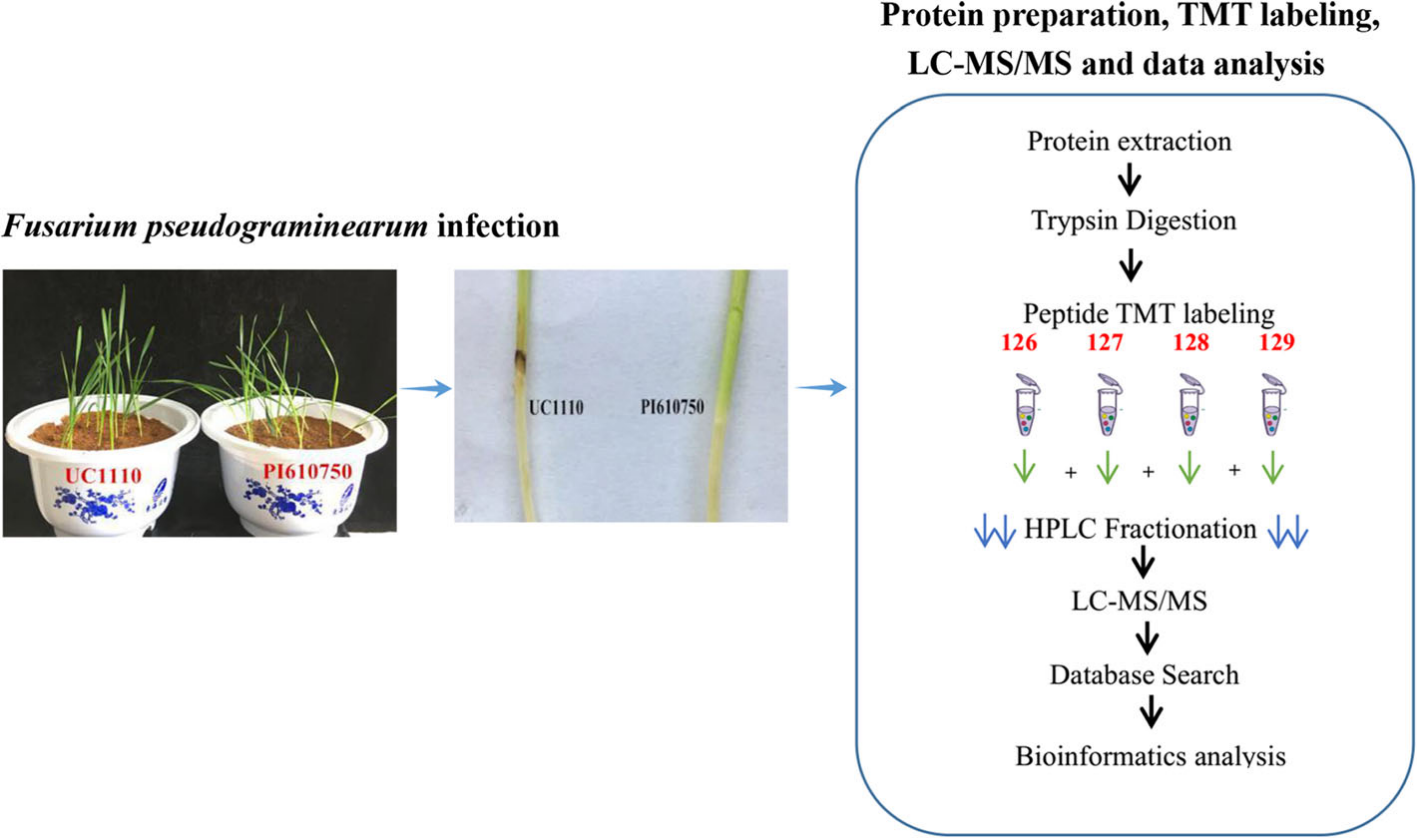
Workflow for the characterization of defense mechanisms of wheat in the response to *F. pseudograminearum* infection using TMT-based quantitative proteomics technology. The stem bases of UC1110 and PI610750 seedlings were inoculated with the colonized grains

We infected one-week-old seedlings with *F. pseudograminearum* from approximately 20 g of millet matrix and used plants at 0 dpi as the control, with untreated 3 dpi seedlings used as the negative control. We collected the stem bases of the two wheat cultivars at 0, 1, 2, and 3 dpi, until symptoms were visible, and then stored the samples at − 80 °C until protein extraction. We performed three biological replicates per treatment (Fig. 9).

### Measurements of plant morphological and physiological parameters

We examined morphology using an Epson Expression 12000XL photo scanner (Seiko Epson Corporation, Suwa, Nagano, Japan) and analyzed morphological parameters using the Win-RHIZO (LA6400XL, Regent Instruments Inc., Quebec, Canada) system, including total root length, total root surface area, total root volume, average root diameter, number of root tips, and number of forks. We collected wheat roots and leaves at different times (0, 1, 2, and 3 dpi). Root activity was determined using the triphenyl tetrazolium chloride modified method, as described by Wang et al. and Cao et al. [61, 62]. We determined the content of total soluble sugar using a sulfuric acid-anthrone method [63, 64] and measured leaf chlorophyll content by spectrophotometry [65]. We measured the activity of enzymes related to stress, POD, SOD, and CAT according to previously described methods [65, 66] and measured the protein content using the Bradford method with bovine serum albumin (BSA) as the standard [67]. We determined MDA content according to previously described methods [65].

### Protein extraction

Protein extraction, trypsin digestion, LC-MS (MS/MS) analysis, and the database search were performed with reference to previously reported methods [68–74], with some modifications. The sample was ground into a cell powder in liquid nitrogen. Then, the cell powder was moved to a 5-mL centrifuge tube. During the next step, we added four volumes of lysis buffer (i.e.,8 M urea, 1% Triton-100, 10 mM dithiothreitol, and 1% Protease Inhibitor Cocktail) to the cell powder. A high-intensity ultrasonic processor (Scientz, Zhejiang, China) was used to perform sonication three times on ice. We removed the remaining debris by centrifugation at 20,000 g at 4 °C for 10 min. Finally, we precipitated the protein with cold 20% trichloroacetic acid for 2 h at − 20 °C. The supernatant was discarded after centrifugation at 12,000 g at 4 °C for 10 min. We washed the remaining precipitate with cold acetone three times. The protein was re-dissolved in 8 M urea and a BCA kit was used to determine the protein concentration according to the manufacturer’s instructions.

### Trypsin digestion

For digestion, we used 5 mM dithiothreitol for 30 min to reduce the protein solution at 56 °C. After that, at room temperature in darkness, the protein was alkylated with 11 mM iodoacetamide for 15 min. Then, we diluted the protein sample by adding 100 mM TEAB until the urea concentration was less than 2 M. Finally, 1:50 and 1:100 trypsin-to-protein mass ratios were used for the first digestion overnight and a second 4 h digestion, respectively.

### LC-MS (MS/MS) analysis

We dissolved the tryptic peptides in 0.1% formic acid (solvent A). Then, peptides were directly loaded onto a homemade reversed-phase analytical column (15 cm length, 75 μm i.d.). At a constant flow rate of 400 nL/min on an EASY-nLC 1000 ultra-performance liquid chromatography (UPLC) system, the gradient of solvent B (0.1% formic acid in 98% acetonitrile) increased from 6 to 23% over 26 min, increased from 23 to 35% in 8 min and climbed to 80% in 3 min, and was then held at 80% for the last 3 min. We subjected the peptides to an NSI source, then tandem mass spectrometry (MS/MS) in QExactiveTM Plus (ThermoFisher, Waltham, MA, USA) was coupled online to the UPLC. The 2.0 kV electrospray voltage was applied. In the Orbitrap, the intact peptides were detected at a resolution of 70,000, and the *m/z* scan range was 350 to 1800 for a full scan. We then selected peptides for MS/MS using NCE set at 28. The fragments were then detected in the Orbitrap at a resolution of 17,500. We conducted a data-dependent procedure, which alternated between one MS scan followed by 20 MS/MS scans with a dynamic exclusion of 15.0 s. We set at 5E4 about the automatic gain control. The first mass was fixed at 100 *m/z.*

### Database search

We used the Maxquant search engine (v.1.5.2.8) to process the resulting MS/MS data. After that, we searched Tandem mass spectra against the UniProt *Triticum aestivum* database, which was concatenated with a reverse decoy database. As a cleavage enzyme, Trypsin/P was allowed up to two missing cleavages. For precursor ions in First search and in Main search, the mass tolerances were set at 20 ppm and 5 ppm, respectively. We set the mass tolerance for fragment ions at 0.02 Da. Oxidation on Met and carbamidomethyl on Cys were specified as a variable modification and a fixed modification, respectively. We adjusted the false discovery rate (FDR) to < 1% and set the minimum score for peptides at > 40. For the protein quantification, we selected TMT 6-plex method. The FDR was set at 0.01, and at least two peptides were required for protein groups quantification. With regard to the protein quantification, protein ratios were calculated through the median of only unique peptides of the protein. We normalized all peptide ratios by the median protein ratio. We used cutoff values of more than 1.50-fold and less than 0.667-fold to identify up-regulated and down-regulated proteins using a *t*-test at *P* < 0.05.

### Bioinformatics analysis

We derived the GO annotation proteome from the UniProt-GOA database (http://www.ebi.ac.uk/GOA/). We used the KEGG database to annotate protein pathways. We analyzed the protein–protein interactions for the identified proteins using the STRING v10.5 database (http://string-db.org) to determine their functions and pathways. We visualized the interaction network from STRING in Cytoscape (https://cytoscape.org/). We used a graph theoretical clustering algorithm [i.e., molecular complex detection (MCODE)] to analyze densely connected regions. MCODE is part of the plug-in toolkit of the network analysis and visualization software Cytoscape.

### Quantitative real-time reverse transcription–polymerase chain reaction

The gene primers were designed by Online Primer 3.0 and are shown in Additional file 7. We extracted the total RNA from the wheat stem bases using the TaKaRa MiniBEST Plant RNA Extraction Kit (TaKaRa, Dalian, China). Reverse transcription of RNA was performed following the kit instructions (Promega Corp., Madison, WI, USA). The detailed method can be found in a previous publication [60]. The reproducibility of the results was guaranteed through three biological replicates. We performed the reactions in a CFX96 Real-Time PCR Detection System (Bio-Rad Laboratories, Inc., Hercules, CA, USA). We analyzed all data using CFX Manager Software (Bio-Rad Laboratories, Inc.). The relative expression levels were calculated using the 2^-ΔΔCT^ method [75]. β-actin was used as an internal control gene.

### Statistical analyses

We performed statistical analyses for morphological results across 10 biological replicates, for physiological and biochemical analyses across four biological replicates, and for proteomic analyses across three biological replicates. We performed analysis of variance using IBM SPSS Statistics 21.0 (IBM Corp., Armonk, NY, USA). Data are presented as means ± standard deviation (SD) values. We determined the statistical significance using Student’s *t*-tests at a *P* < 0.05 threshold.

## Supplementary Information

**Additional file 1: Table S1.** GO functional enrichment of 366 DEPs.

**Additional file 2: Fig. S1.** Distribution of all differentially expressed proteins in the KEGG pathway.

**Additional file 3: Table S2.** KEGG pathway enrichment of C1.

**Additional file 4: Table S3.** KEGG pathway enrichment of C2.

**Additional file 5: Table S4.** KEGG pathway enrichment of C3.

**Additional file 6: Table S5.** Protein-protein interaction network.

**Additional file 7: Table S6.** Primer sequence.

## Data Availability

All data analyzed in this study are included in this published article and its additional files. The mass spectrometry proteomics data have been deposited to the ProteomeXchange Consortium via the PRIDE partner repository (http://www.ebi.ac.uk/pride) with the dataset identifier PXD023314.

## Abbreviations

TMT: Tandem mass tag
dpi: Days post inoculation
DEPs: Differentially expressed proteins
FCR: Fusarium crown rot
QTLs: Quantitative trait loci
CK: Untreated seedlings at 3 dpi
MDA: Malondialdehyde
SOD: Superoxide dismutase
POD: Peroxidase
CAT: Catalase
GO: Gene ontology
KEGG: Kyoto encyclopedia of genes and genomes
qRT-PCR: Quantitative real time–polymerase chain reaction
ProDH: Proline dehydrogenase
GSTs: Glutathione S-transferases
AOS: Allene oxide synthase
JA: Jasmonic acid
NADH-GOGAT: NADH-dependent glutamate synthetase
PR proteins: Pathogenesis-related proteins
SAR: System-acquired resistance
UPLC: Ultra-performance liquid chromatography
FDR: False discovery rate
