# Supplementary figures and images for: TMT-based quantitative proteomic analysis reveals defense mechanism of wheat against the crown rot pathogen Fusarium pseudograminearum
Source: BMC Plant Biol. 2021 Feb 8;21:82. doi: 10.1186/s12870-021-02853-6 (PMC7869478; doi:10.1186/s12870-021-02853-6)

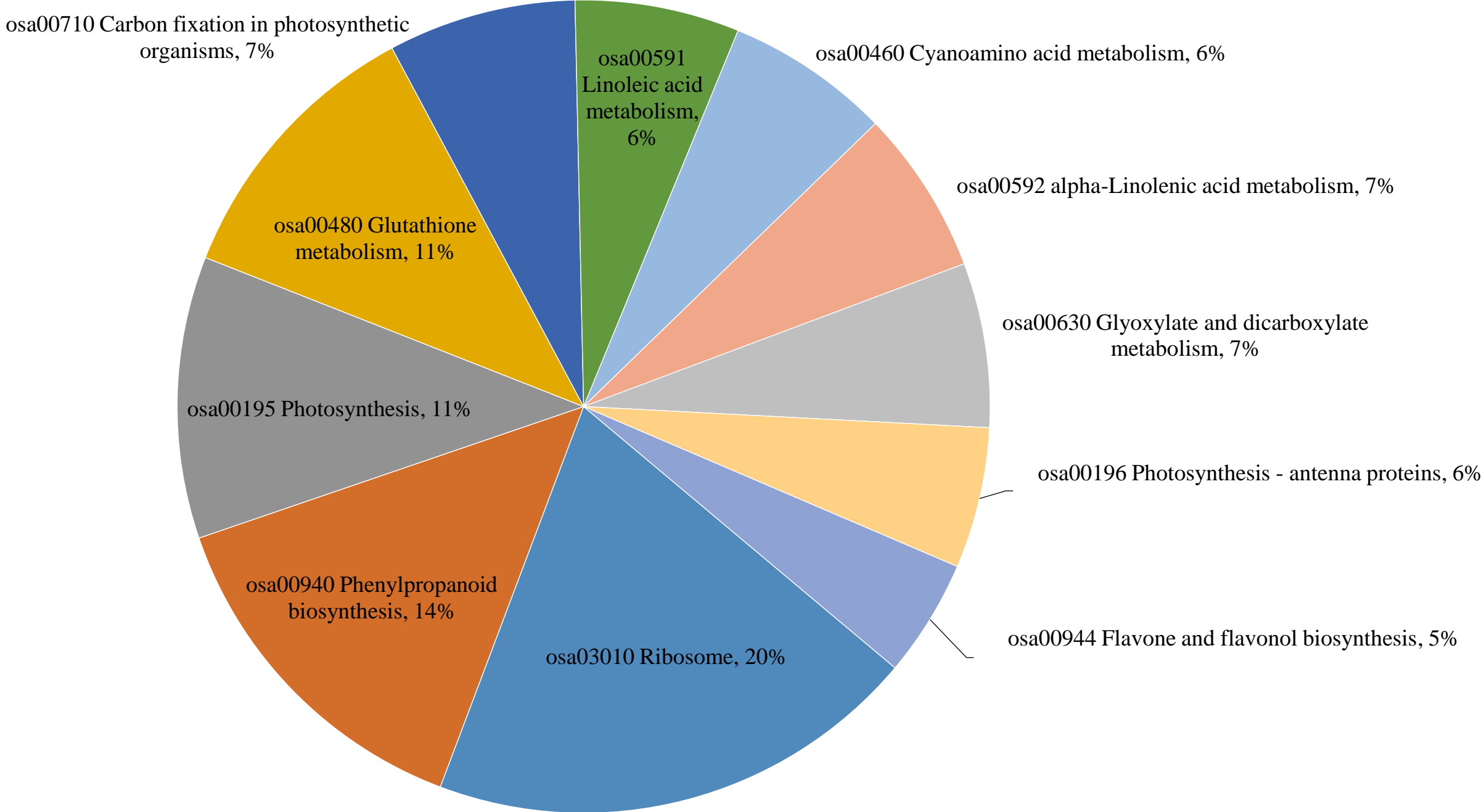

Figure S1. Distribution of all differentially expressed proteins in the KEGG pathway

Supplement: Supplementary file 2 — Additional file 2: Fig. S1. Distribution of all differentially expressed proteins in the KEGG pathway. [file 12870_2021_2853_MOESM2_ESM.pdf]
